# Enhancing laparoscopic simulator performance with eye-tracking video feedback: a mixed-methods pilot study

**DOI:** 10.3389/fsurg.2026.1780289

**Published:** 2026-03-18

**Authors:** Ninos Oussi, Gabriel Sandblom, Lars Enochsson

**Affiliations:** 1 Centre for Clinical Research Sörmland, Uppsala University, Eskilstuna, Sweden; 2 Department of Surgery, Södersjukhuset, Stockholm, Sweden; 3 Department of Clinical Science and Education, Södersjukhuset, Karolinska Institutet, Stockholm, Sweden; 4 Department of Diagnostics and Intervention, Surgery, Umeå University, Umeå, Sweden; 5 Division of Orthopedics, Department of Clinical Science Intervention and Technology (CLINTEC), Karolinska Institutet, Stockholm, Sweden

**Keywords:** eye-tracking, feedback, gaze, Simball Box, virtual reality

## Abstract

**Background:**

The utility of eye-tracking combined with video-feedback to enhance laparoscopic simulator training remains unexplored. This mixed-methods pilot study aimed to evaluate whether visualising gaze patterns during video feedback improves simulator performance and training experience.

**Materials and methods:**

Ten surgical residents were randomised into an experimental group (receiving gaze-overlay video feedback) or a control group (standard video feedback). Participants performed standardised tasks in the Simball Box simulator. Quantitative performance metrics (time, error rates) and eye-tracking data (gaze patterns via Tobii Pro) were analysed. Qualitative data were collected through pre- and post-questionnaires and a subsequent group interview to assess the perceived value of the training.

**Results:**

Although 90% of residents had prior experience, the majority (60%) found the specific tasks more challenging than anticipated. Subjective ratings of feedback value did not differ significantly between groups. However, objective heat map analysis revealed that the experimental group adopted significantly more focused gaze patterns. Qualitative interviews underscored that while technological feedback is valuable, it is most effective when paired with expert mentorship.

**Conclusion:**

This pilot study suggests that eye-tracking feedback may facilitate more efficient visual strategies during laparoscopic training. While the experimental group demonstrated initial performance gains, larger cohorts are needed to statistically validate these findings. The study further emphasises that for simulation technology to be meaningful, it must be integrated into a structured curriculum with expert guidance.

## Introduction

Simulator training is now a cornerstone of surgical residency curricula globally, with proven benefits for operative performance across diverse specialties (1–9) While devices ranging from simple box trainers (10) to sophisticated Virtual Reality (VR) (11) systems have effectively shortened learning curves, current models possess a critical limitation (12–14) Standard feedback metrics primarily quantify motor skills—such as instrument path length and time—yet fail to address the cognitive component of visual attention. Novice surgeons frequently exhibit inefficient gaze patterns compared to experts, however, traditional training rarely addresses where a trainee looks, focusing only on how they move.

Eye-tracking technology, though well-established in the gaming industry (15, 16) offers a unique modality to objectively measure attention and focus in medical training (17–22) Nevertheless, high costs and a lack of validation have hindered widespread clinical implementation (12). For virtual environments to gain full acceptance, simulators must be validated in clinical trials and proven to accurately reflect real-life clinical practice (13). Consequently, before such technology can be justified in routine curricula, its feasibility and pedagogical value must be rigorously tested (12).

To our knowledge, the utility of combining eye-tracking with gaze-overlay video feedback to enhance laparoscopic simulator performance remains unexplored. While eye-tracking has shown promise for skills assessment, further research is needed to establish its value as a training tool specifically designed to improve performance (14).

This mixed-methods pilot study addressed two primary objectives:

1. To assess whether visualising gaze patterns during video feedback improves objective simulator performance.
2. To evaluate surgical trainees' motivation and perceptions regarding eye-tracking as an educational tool.

## Materials and methods

### Participants and procedures

**Figure 1:**
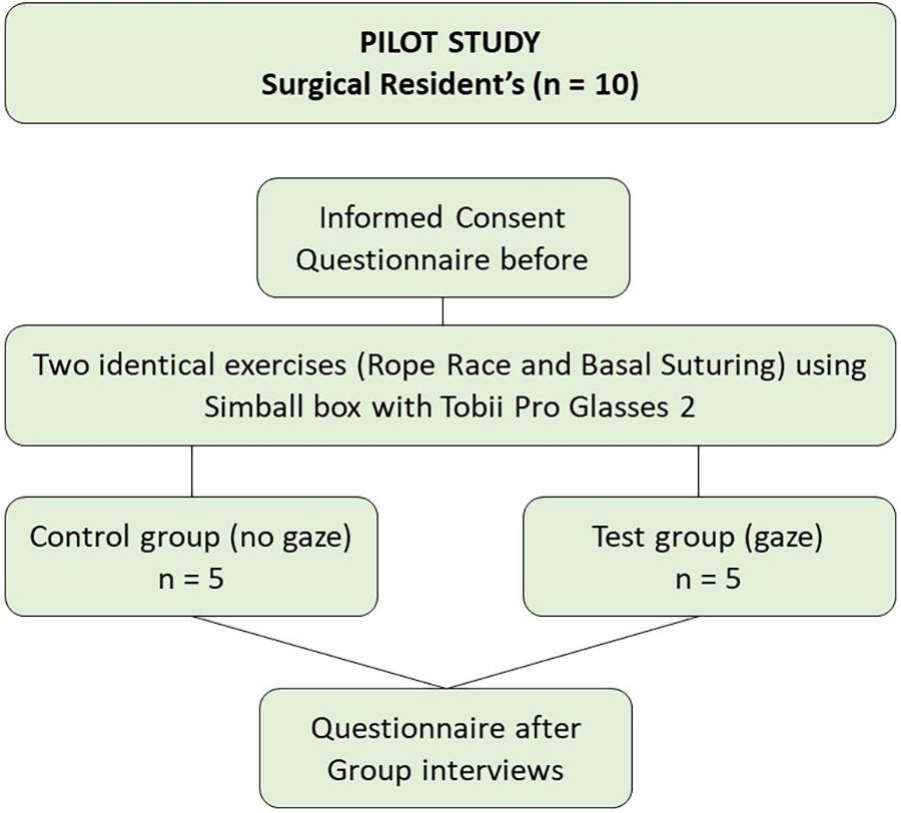
Pilot study flowchart.

Ten surgical residents (3 females, 7 males; mean age 32.9 years) from the Department of Surgery and Urology, Mälar Hospital, Eskilstuna, Sweden, volunteered for this mixed-methods pilot study. To ensure that the cohort reflected a real-world residency setting, inclusion was open to all surgical residents without strict exclusion criteria, aiming specifically to capture a group with limited prior simulation experience (Figure 1).

**Figure 2:**
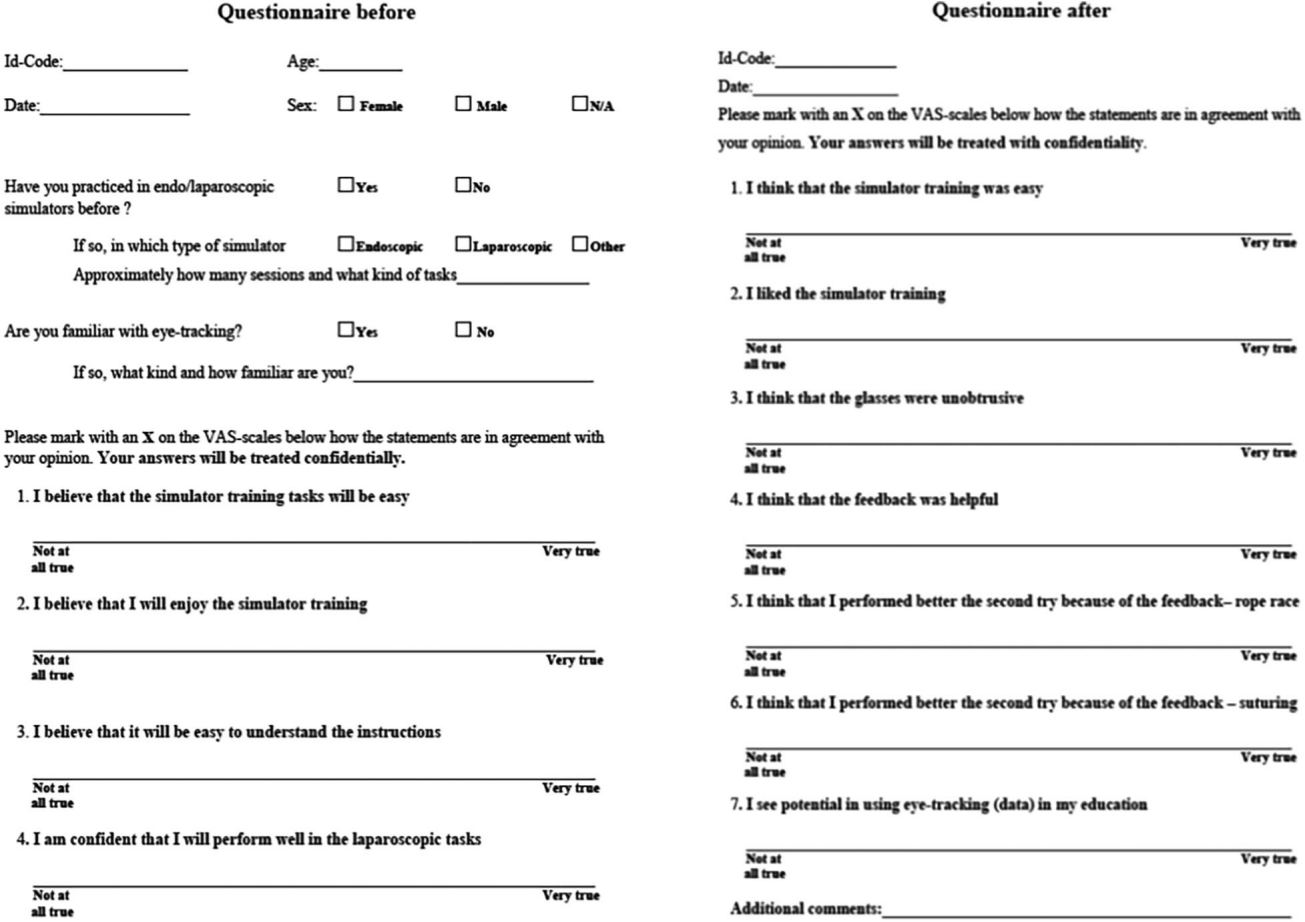
Questionnaires *before*, and *after* task performances.

Following informed consent, participants completed a baseline questionnaire covering demographics, expectations, and previous simulation exposure (Figure 2).

Randomisation was achieved using sealed opaque envelopes, allocating residents to either the experimental group (*n* = 5) or the control group (*n* = 5). The randomisation process aimed to balance variations in prior experience between the groups, mitigating the limitations of the small sample size.

### Simulator tasks and intervention

Both groups received standardised verbal instructions and viewed expert-benchmark videos on the Simball Box simulator (Surgical Science Sweden AB) (Table 1). The core intervention centred on the feedback mechanism: the experimental group reviewed their performance with a gaze overlay—a visual cue designed to facilitate mimicry of expert visual strategies—whereas the control group viewed the replay without gaze markers.

**Table 1:** (A) Benchmark expert values of the Simball Box tasks. (B) Number of subjects who improved performance after second task.

A	Rope Race		Basal Suturing	
Linear Distance (cm)	220		210	
Angular Distance (cm)	32		32	
Total Time (s)	60		63	
B	Rope Race		Basal Suturing	
Test	1^st^	2^nd^	1^st^	2^nd^
Test Group (*n*)	2	5	3	5
Control Group (*n*)	5	5	2	5
Total (*n*)	7	10	5	10

Participants performed two standardised tasks, consecutively: *Rope Race* (threading a rope through rings) and *Basal Suturing* (surgical knot tying). Performance metrics, including time and angular/linear distance, were recorded automatically by the Simball Box software (Table 2).

**Table 2:** Simball Box metrics for both tasks per subject, respectively, with and without gaze.

Test-person	Gaze	Metrics	Rope race 1	Rope race 2	Rr2-Rr1	Suture 1	Suture 2	S2-S1
1	No	Linear distance [cm]	402	390	−12	640	808	168
		Angular distance [rad]	67	74	7	105	138	33
		Total time [s]	141	139	−2	209	302	93
2	Yes	Linear distance [cm]	447	508	61	826	724	−102
		Angular distance (rad)	79	77	−2	122	145	23
		Total time (s)	159	192	33	276	223	−53
3	No	Linear distance (cm)	408	399	−9	296	268	−28
		Angular distance (rad)	73	68	−5	56	95	39
		Total time (s)	108	134	26	71	110	39
4	Yes	Linear distance (cm)	536	370	−166	944	905	−39
		Angular distance (rad)	80	85	5	162	245	83
		Total time (s)	166	151	−15	309	229	−80
5	No	Linear distance (cm)	461	372	−89	545	329	−216
		Angular distance (rad)	68	48	−20	303	-	-
		Total time (s)	123	114	−9	196	206	10
6	Yes	Linear distance (cm)	323	309	−14	626	582	−44
		Angular distance (rad)	79	71	−8	108	121	13
		Total time (s)	159	135	−24	198	206	8
7	Yes	Linear distance (cm)	399	414	15	726	746	20
		Angular distance (rad)	63	48	−15	160	189	29
		Total time (s)	133	134	1	158	179	21
8	Yes	Linear distance (cm)	299	188	−111	559	650	91
		Angular distance (rad)	38	27	−11	155	171	16
		Total time (s)	108	67	−41	98	159	61
9	Yes	Linear distance (cm)	517	290	−227	1131	904	−227
		Angular distance (rad)	61	53	−8	109	132	23
		Total time (s)	163	88	−75	224	136	−88
10	No	Linear distance (cm)	555	315	−240	937	612	−325
		Angular distance (rad)	75	65	−10	111	167	56
		Total time (s)	163	88	−75	224	136	−88

### Eye-tracking and equipment

Gaze data were captured using Tobii Pro Glasses 2 (Tobii AB), wearable eye-tracking glasses equipped with a scene camera recording the visual field. The Tobii Pro Glasses Analyzer software (v.1.34.1003) was used to visualise gaze data, plot areas of interest, and generate heat maps.

### Focus group interviews

The focus group interview session was scheduled two weeks after finalising the randomisation process, and aimed to:

1. Understand needs and motivations of the residents.
2. Discuss the pilot study and the feedback concept.
3. Explore opportunities for eye-tracking in medical applications (Table 3).

**Table 3:** Questions related to the focus group interviews.

Background and motivation	
1.	Background, laparoscopic/simulator experience
2.	What do you find (a) difficult and (b) engaging/rewarding, at work?
3.	What motivates/inspires you in your education and work?
4.	When learning a new physical skill or a new procedure—what's your approach?
5.	Do you have any tricks or gimmicks to learn?
6.	Recount a moment when you felt having a good learning-strategy
7.	How much do you reflect on your own behaviour? Do you watch a replay of your own performance? Why?
8.	Which factors are important in education (laparoscopy)? Why?
Laparoscopic simulators	
9.	How does it usually work when you practice on simulators?
	(a) Practice in groups? How many participants each time? (b) What do you prefer? Practice in solitude or in discussion with others/teachers? (c) Why?
10.	What challenges do you see in using Glasses 2 within your research? Why?
11.	What pros do you see with visual training? Why?
12.	Combination med verbal instructions? Why?
Expectations	
13.	Share your expectations and knowledge about eye-tracking?
14.	Do you have anything to add?

### Qualitative analysis

Qualitative data from focus group interviews (Table 3) underwent thematic analysis to identify recurring patterns regarding training motivation, obstacles (e.g., clinical interruptions), and attitudes toward the technology. Subjective experiences were quantified using a Visual Analogue Scale (VAS) (23) converted to percentage values.

### Statistical analysis

Statistical analysis was performed using JMP® Pro version 14.0.0 (SAS Institute Inc., USA), with significance set at *p* < 0.05. Due to the sample size and non-parametric nature of the data, results are presented as medians and ranges. Group comparisons were conducted using Wilcoxon/Kruskal–Wallis tests, whilst categorical data were analysed using Chi-squared or Fisheŕs exact tests. Fit-line analysis was used to correlate training experience with simulator performance.

## Results

### Demographics and prior experience

Ninety percent of the participants reported prior experience with laparoscopic simulators, with self-reported practice times ranging from 0.5 to 10 h. Regarding the study tasks, 60% of the participants rated them as more difficult than anticipated.

### Video-feedback assessment

Subjective ratings of the video-feedback were generally positive, ranging from 48% to 93% on the VAS. There were no statistically significant differences between the groups regarding the perceived benefit of the feedback.

### Eye-tracking analysis

**Figure 3:**
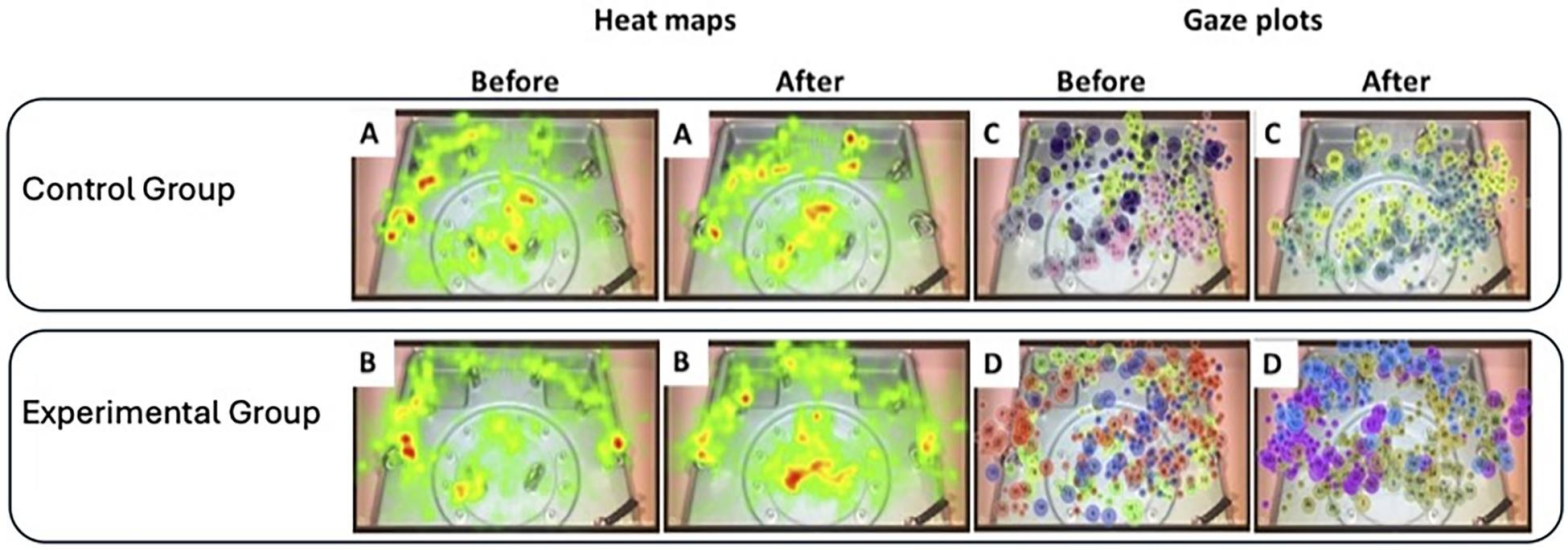
Comparison of heat maps (left), and gaze plots (right), before and after feedback of control group **(A,C)**, and test group **(B,D)**, performing *rope race*.

Gaze data are visualised as gaze plots and heat maps (Figure 3). Residents in the experimental group (gaze overlay) demonstrated improved performance compared to the control group (Table 2). While eye-tracking data were inconclusive for visualising performance differences in the suturing task, heat map analysis for the *Rope race* task indicated that the experimental group exhibited significantly more focused and concise gaze patterns compared to controls (Figure 3).

### Focus group interviews

#### Motivation and current training habits

Residents expressed high motivation to acquire new skills, often dedicating personal time to assist in surgical procedures (Table 4). The preferred learning method described by participants involved a stepwise approach: reading about a procedure, observing a colleague, and subsequently performing the procedure under supervision (Table 5).

**Table 4:** (A) Residents' *dislikes/likes* regarding interruption while conducting the tasks and surgery in general, and (B) *benefits/drawbacks* identified when executing the focus group session.

Dislikes	Likes
Answer pager and telephone interrupting	Practical work tasks it is stimulating to work with hands-on tasks
Administrative work and routine tasks not stimulating	Perform surgery! The most fun task, challenging, it is a craftsmanship
Work in the emergency room high pressure, stressful	Reception of patients and smaller operations are interesting
Stress not being able to do all that is needed in one day work never ends	Acquire new knowledge room for development
Benefits	Drawbacks
Get direct input from users	Time aspect for participants
Mutual understanding between customer and company	Requires effort from participants
Enhances interest	
Casual to perform	

**Table 5:** Quotations from the residents during the focus group interviews.

Residents' comments when:	Resident #1	Resident #2	Resident #3
Acquiring new skills:	It's important with an experienced supervisor.	I learn more when I receive direct feedback from experienced colleague when performing a task.	-
Discussing simulator training:	I don't use it (authors note: the simulator) regularly. I already stay after working hours to participate in operations.	-	There's no time to practice. I can't let my patients wait.
Using eye-tracking in surgical training:	I would like to see a video of both the hands and the gaze pattern combined, because it's important in laparoscopic surgery.	-	There's potential to use it in the practical training, perhaps to make training more efficient, because we don't have much time.
	Simulator training should be scheduled in our study plan. It's hard to find time to practice otherwise.		

#### Barriers to training

Participants identified the lack of dedicated practice time as a significant barrier. Despite the availability of simulators, practicing during working hours was often perceived as inappropriate due to competing clinical duties. Consequently, residents emphasised the necessity of integrating simulation training formally into the curriculum to compensate for the lack of spare time.

#### Perceptions of eye-tracking

Participants agreed that eye-tracking has the potential to enhance training efficiency, a crucial factor given their demanding schedules. Suggested applications included team training exercises, emergency simulations, and tools for self-reflection. However, participants noted that the pilot study's scope was too narrow to demonstrate a definitive effect. They suggested that longitudinal comparisons (e.g., over 30 attempts) and combining gaze visualisation with hand movement recordings would yield more valid data.

#### Supervision

Residents highlighted the critical importance of having access to experienced supervisors and receiving instant feedback during training.

## Discussion

The integration of eye-tracking technology has evolved significantly in recent years across several domains (18–20). In medical education, eye-tracking has shown promise in facilitating debriefing and enhancing educational outcomes, although further feasibility studies are warranted (12). A recent systematic review provided a comprehensive overview of existing research, aiming to guide future studies and clarify the tools available for gaze tracking (14).

Established literature confirms that expert and novice gaze patterns differ significantly (21, 22, 24). Our findings align with previous studies, demonstrating distinct differences in gaze patterns between the experimental and control groups (24–26). Specifically, the experimental group in our study exhibited more focused and concise gaze patterns in the *Rope Race* task, suggesting that visual feedback helped them approximate expert behaviour more rapidly. While the application of eye-tracking to study teamwork and decision-making in emergency care appears promising, further targeted and rigorous research is required to substantiate these potential benefits (27).

Eye-tracking has also been explored as a tool to elucidate human learning processes, helping educators better understand the cognitive mechanisms of trainees (28). Moreover, structured and cognitively focused training methods have been shown to significantly improve training outcomes (28). Consequently, the implementation of standardised proficiency metrics could facilitate the development of local curricula targeting psychomotor skills through VR-based laparoscopic training (29). In a recently published systematic review within the field of medical education, eye-tracking methodology was presented as having contributed significantly to the assessment, feedback practices, and training used in a clinical setting (18).

Furthermore, feedback is a cornerstone of effective learning, particularly when mastering new medical procedures (30, 31). In our study, residents who received eye-tracking–based feedback demonstrated superior simulator performance compared to those who did not. It is well-recognised that individualised instructor feedback enhances performance in simulated laparoscopic training (30). Crucially, since all participants in our study—regardless of group allocation—received comparable verbal feedback from an instructor, the observed performance advantage in the experimental group can be attributed to the addition of the gaze-overlay video feedback. This suggests that visualising one's own gaze provides a unique, additive value to standard mentorship.

### Evaluation of focus group interviews

The focus group session (Table 4) revealed several key insights regarding the traineeś perceptions. Immediate, performance-related feedback was regarded as the most beneficial aspect of training. Furthermore, the efficacy of individualised feedback in enhancing outcomes during laparoscopic simulation training has previously been demonstrated by Ahlborg et al. (30).

The group session facilitated awareness among participants and potential users regarding the application of eye-tracking in training, through open discussions of needs, motivations, and experiences. Moreover, focus group interviews have been demonstrated to elicit higher levels of engagement and stimulation among participants compared to other group interview methodologies (32). Moreover, the session appeared to increase the test subjects' interest in eye-tracking applications, given their limited prior familiarity with the technology. A willingness to expand knowledge—exemplified by explicitly affirming the potential of eye-tracking in their education—was associated with greater motivation. The educational value of eye-tracking devices in medical training has also been emphasised in prior scholarly reviews (18).

With respect to the limitations, the group session highlighted challenges related to participant recruitment. Within the surgical department where the session was conducted, residents faced considerable workload demands and were unable to disengage from pagers and telephones. These circumstances may have resulted in a suboptimal focus group composition and potentially influenced the dynamics of group interaction. The methodological considerations of focus group interviews, along with common difficulties encountered in their implementation, have previously been discussed by Parker and Tritter (33).

### Limitations

Although the limited sample size of this pilot study precludes definitive statistical conclusions, the findings provide valuable preliminary insights into the potential utility of gaze-based feedback. The results from the focus group interviews should be interpreted with caution, as the small number of participants and interruptions from clinical duties may have influenced the responses. Furthermore, data from the Simball Box and questionnaires revealed considerable individual variability, likely attributable to differences in personality traits and prior simulation experience.

Another limitation concerns the ecological validity of the setting; disruptions caused by ongoing clinical duties may have introduced stressors that negatively affected participants' performance. Additionally, we solely tested eye-tracking feedback using the Simball Box simulator. Whether these results can be extrapolated to other simulated environments or real-life clinical training remains uncertain and requires further investigation.

## Conclusion

This pilot study suggests that eye-tracking with gaze-overlay feedback can accelerate the acquisition of expert-like visual strategies in laparoscopic training. While technical proficiency requires repeated practice, visualising gaze patterns appears to promote greater efficiency and focus even in early learning stages. However, technology alone is not a panacea; our qualitative findings underscore that such tools are most effective when integrated into a structured curriculum supported by expert mentorship. Future research should validate these findings in larger cohorts and explore the transferability of gaze-training to the operating room.

## Data Availability

The raw data supporting the conclusions of this article will be made available by the authors, without undue reservation.
